# Gut inflammation and tumorigenesis: every site has a different tale to tell

**DOI:** 10.1007/s11739-023-03320-w

**Published:** 2023-05-30

**Authors:** Alessandro Vanoli, Paola Parente, Matteo Fassan, Luca Mastracci, Federica Grillo

**Affiliations:** 1 Anatomic Pathology Unit, Department of Molecular Medicine, University of Pavia, Via Carlo Forlanini 16, 27100 Pavia, Italy; 2 Anatomic Pathology Unit, IRCCS San Matteo Hospital, Pavia, Italy; 3 Unit of Pathology, Fondazione IRCCS Ospedale Casa Sollievo Della Sofferenza, San Giovanni Rotondo, FG Italy; 4 Surgical Pathology Unit, Department of Medicine (DIMED), University Hospital of Padua, Padua, Italy; 5 Veneto Institute of Oncology IOV-IRCCS, Padua, Italy; 6 IRCCS Ospedale Policlinico San Martino, Genoa, Italy; 7 Anatomic Pathology, Department of Surgical Sciences and Integrated Diagnostics (DISC), University of Genova, Genoa, Italy

**Keywords:** Cancer, Celiac disease, Esophagitis, Gastritis, Inflammatory bowel disease

## Abstract

Gut inflammation has been correlated with cancerogenesis by disrupting gastrointestinal homeostasis. Numerous chronic inflammatory disorders of the tubular gastrointestinal tract (e.g., gastroesophageal reflux disease, *Helicobacter pylori*-induced and autoimmune chronic gastritis, celiac disease, and inflammatory bowel diseases) have been variably associated with an increased neoplastic risk. Gastrointestinal inflammation-induced neoplasms include epithelial tumors (esophageal squamous cell carcinoma and adenocarcinoma, gastric adenocarcinoma and neuroendocrine tumors, small bowel adenocarcinoma and neuroendocrine tumors, and colorectal cancer) and lymphomas (such as gastric marginal zone lymphomas and enteropathy-associated T cell lymphoma). In the last decades, numerous studies have investigated the pathogenetic mechanisms and the microenvironmental/microbiome changes that trigger genetic and/or epigenetic alterations eventually leading to tumorigenesis, often through a histologically recognizable inflammation-dysplasia-carcinoma cancerogenic sequence. In the present review, an overview of the current knowledge on the links between inflammatory diseases and neoplasms of the tubular GI tract, applying a site-by-site approach, is provided.

## Introduction

The link between inflammation and cancer was first postulated by the father of modern pathology, Rudolph Virchow, who, in 1863, described inflammatory cell infiltration within cancer growths. This led to the hypothesis that there was indeed a correlation between inflammation and carcinogenesis and that cancer could originate in sites of chronic inflammation and this has been proven by numerous research groups, and in various sites, over time [1].

Gut inflammation is known to disrupt gastrointestinal (GI) homeostasis and several chronic inflammatory disorders of the tubular GI tract (esophagitis, gastritis, enteritis and colitis) have been associated with an increased risk of developing solid and/or hematolymphoid neoplasms. Although there is evidence that some gut inflammatory disorders are caused by either infectious agents [e.g., *Helicobacter pylori* (*HP*)-related gastritis] or chemical injury (e.g., reflux esophagitis), many of them have an autoimmune or immune-mediated etiopathogenesis. In the last decades, numerous studies have investigated the biological mechanisms and microenvironmental/microbiome changes that trigger genetic and/or epigenetic alterations eventually leading to tumorigenesis in such conditions. In this review, we provide an overview of current knowledge on the links between inflammatory diseases and neoplasms of the tubular GI tract, applying a site-by-site approach.

## Role of inflammation in the development of esophageal cancers

Two main types of epithelial cancers can affect the esophagus, namely squamous cell carcinoma (ESCC) and adenocarcinoma (EAC), which altogether cover more than 95% of esophageal malignancies. ESCC and EAC differ both for esophageal location (with involvement of middle and upper third of the esophagus by ESCC versus lower third and gastro-esophageal junction by EAC) and geographic distribution. EAC has in fact continued to increase in incidence in western countries in the last fifty years, thus overwhelming ESCC, which, however, still represents the predominant subtype globally.

Different causative agents have been correlated with cancer development in the esophagus, spanning from cigarette smoke, alcohol and/or hot beverage consumption, diet, infectious agents and gastroesophageal reflux disease (GERD). A major effort has been made by researchers to understand the molecular events which, following the exposition of esophageal epithelium to causative agents, lead to cancer development. In this context, a major role seems to be played by inflammation, both per se [2] and in combination with alterations of the microbiota [3].

Tobacco and alcohol consumption, which are the major risk factors for ESCC, promote cancer development via acetaldehyde which has a carcinogenic effect by forming DNA adducts and altering genes [4]. Acetaldehyde is a constituent of tobacco smoke and the first metabolite of ethanol; the combined use of tobacco and smoke act synergistically in the cascade of events driving progression from the normal squamous epithelium to pre-invasive and invasive neoplasia [5]. Moreover, tobacco and alcohol directly act on the inflammatory-immune system inducing production of several cytokines [i.e., interleukin (IL) 1–6–8] and forming free radicals (reactive oxygen and nitrogen species) which lead to oxidative stress and activation of the nuclear factor kappa B (NF-kB) family [6]. This creates a pro-inflammatory state responsible of inflammation and contributing to carcinogenesis [4].

The relationship between GERD and inflammation has been extensively studied in the last century. A major role is played both by gastric and bile acid in the gastro-esophageal refluxate. For several years the proposed model to explain the link between GERD and inflammation, was through direct damage of the superficial epithelial layer caused by acid refluxate, with necrosis and acute inflammation (neutrophils and eosinophils) permeating the squamous epithelium. This damage, in addition to the recruitment of inflammatory cells, is responsible for epithelial proliferation (manifesting as basal cell hyperplasia and papillary elongation) aimed at repairing, by substitution, the damaged surface epithelial cells. Furthermore, acid also damages the intercellular junctions, causing an increase in epithelial permeability (manifesting with dilatation of intercellular spaces) which enable the hydrogen ions to spread between epithelial cells [7]. In contrast with this hypothesis, in which damage starts from surface and proceeds to the deep portions of the mucosa, a rat model for reflux esophagitis suggests that inflammation starts in the submucosa, with recruitment of T-lymphocytes, mediated by release of pro-inflammatory cytokines (IL-8) by the squamous epithelium, and successively proceeds toward the surface [8]. This cytokine-mediated model with initial T-lymphocyte recruitment has also been confirmed in humans [9] and T-lymphocytes have been demonstrated to be significantly increased in biopsies of patients with both non-erosive and erosive reflux disease compared to healthy controls, while B-cells, Langerhans cells, Natural Killer cells and macrophages play a marginal role [10].

Similarly to exposure of the esophageal squamous epithelium to alcohol and smoke, the generation of reactive oxygen species, which stabilize and activate hypoxia-inducible factor (HIF)-2α, are linked to the pro-inflammatory IL cascade in GERD [11]. This cascade is responsible for the oxidative stress status which contributes to the development of Barrett’s esophagus (BE) and EAC [12].

Under the pressure of prolonged acid refluxate injury and inflammation, the esophageal squamous stratified epithelium is replaced by simple columnar epithelium thus starting the cascade of morphological and molecular events that from BE drive to EAC and Gastro-Esophageal Junction adenocarcinoma (GEJA) via low-grade dysplasia (LGD) and high-grade dysplasia (HGD) through an inflammation-dysplasia-carcinoma cancerogenic sequence. In each step of this cascade, inflammation plays an important role and an increase of T-cells, B cells, macrophages and dendritic cells, has been reported in BE and EAC/GEJA. Moons et al. have demonstrated that BE shows a predominant humoral immune response (Th2) while GERD shows a more pronounced cellular immune response (Th1) [13]. In this study, immunohistochemical analysis for the principal Th1 (macrophages and CD8+ T lymphocytes) and Th2 (plasma cells and mast cells) effector cells was performed, showing an increase in Th2 effector cells in BE with equal number of Th1 effector cells compared to GERD as well as a predominant expression of IgG and IgE by plasma cells. This shift toward a more humoral immune response in BE is associated with progressive depression of the cell-mediated immunity and this, on the one hand is correlated with angiogenesis while, on the other, causes a reduction of immune surveillance; these two sides of the coin are both involved in tumoral progression [13]. These observations have been confirmed and detailed in the study by Kavanagh et al. who, in addition to the Th1 vs Th2 profile shift from GERD to BE, demonstrated a significantly lower number of activated T-cells in EAC, with an increase in both pro and anti-inflammatory cytokines, probably leading to a mixed inflammatory profile in the final steps of the neoplastic cascade [14]. The recent studies by Lagisetty et al. and Sundaram et al. shed greater light on the dynamic changes in the immune landscape from normal esophagus, to BE, LGD- and HGD, and EAC using a sequential multiplex immunohistochemistry platform [15, 16]. Both studies have demonstrated a progressive increase of CD8+ T cells in the different steps of the neoplastic cascade, leading to decreased cytotoxic effector cells and an immunosuppressive microenvironment in EAC.

Another fundamental protagonist in the esophageal microenvironment, which can influence inflammation and is emerging as a potential driver of oncogenesis [17], is represented by the microbial flora. The microbial population changes in pathological conditions (GERD and BE) with respect to the normal healthy subject, with an increase in gram-negative bacteria. These gram-negative bacteria produce lipopolysaccharides (LPS) which cause high levels of pro-inflammatory cytokines via NF-κB activation with a simultaneous increase in IL-1β, IL-6, IL-8 and tumor necrosis factor (TNF) along the spectrum of GERD, BE and EAC. Altogether, the alteration in the microbiome of BE may lead to EAC by triggering chronic inflammation and propagating the inflammatory cascade [3].

## Inflammation and gastric neoplasia

**Fig. 1:**
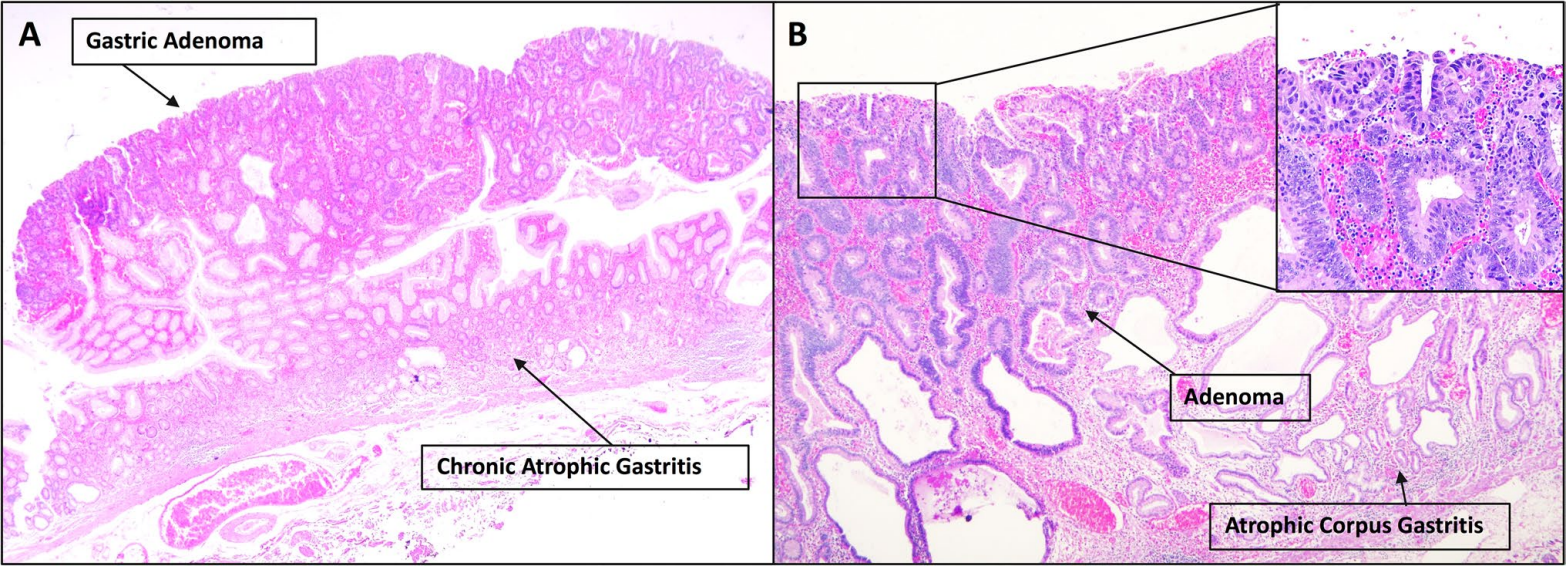
A histologic example of a gastric adenoma arising in the context of chronic atrophic gastritis of the oxyntic mucosa. Note in **A** the polypoid appearance of the adenoma and in **B**, at higher magnification, the inflammation with atrophy of the oxyntic mucosa and the high-grade cytoarchitectural features of the dysplastic adenomatous proliferation (**A**, **B** hematoxylin and eosin)

Longstanding mucosal inflammation is the main cause of the cancerogenic cascade leading to sporadic gastric neoplasia. The etiological agents responsible for gastritis, described in the Kyoto Classification, are environmental and host-related [18]. The environmental etiology is far more common, and includes both transmissible and non-transmissible agents. The epidemiological impact of the host-related category is significantly lower, and it includes different etiologies, most of which are immune-mediated disorders. Environmental and host-related etiologies may also overlap, as in the case of autoimmune gastritis triggered by *HP* infection. Both etiologic models result in the atrophic remodeling of the native gastric mucosa and chronic immune-system stimulation, promoting epithelial neoplastic lesions, following the inflammation-dysplasia-carcinoma sequence (Fig. 1), as well as gastritis-related lymphomas [19].

*HP* is both the most common and the best understood gastric carcinogen [20]. The prevalence of *HP* infection differs with age, geographic regions (up to 80% of middle-aged adults in the developing countries), socio-economic status, education level, living environment and occupation. Although the majority of *HP* infected patients remain asymptomatic, essentially all develop chronic inflammation, approximately 10% develop peptic ulcer disease, 1–3% progress to GC, and 0.1% develop mucosa-associated lymphoid tissue (MALT) lymphoma [21]. A positive association between consumption of salt-cured foods (fish, meat, vegetables), tobacco smoke, alcohol consumption, long-term use of proton pump inhibitors and increased risk of gastric cancer (GC) is described, which is stronger in subjects with *HP* infection [19].

*HP* produces a variety of virulence factors that may dysregulate host intracellular signaling pathways leading to neoplastic transformation. In particular, the *HP*-related toxin *CagA (*cytotoxin-associated gene A): (1) enhances proliferation by various molecular pathways; (2) it disrupts tight junctions leading to loss of polarity; (3) it interferes with oncogenes (such as p53 and Runt-related transcription factor3—RUNX3); (4) it activates the oncogenic RAS pathway by attenuating miRNA let-7 expression [21]. On the other hand, vacuolating cytotoxin A (*VacA)* is involved in epithelial tight junction disruption, apoptosis and suppressed T lymphocyte activation and proliferation by activation of Bax (Bcl 2 associated X protein) [21].

*HP* up-regulates pro-inflammatory cytokines such as IL-1, IL-6, IL-8, TNF-α, NF-κB, and it induces the generation of intracellular reactive oxygen species (ROS) and reactive nitrogen species (RNS) by host gastric epithelial cells and inflammatory cells. Moreover, *HP* may lead to hypermethylation of O6-methylguanine DNA methyltransferase (MGMT), with secondary silencing of many tumor suppressor genes such as Trefoil factor family 2, E-cadherin, p16, mismatch repair gene (*hMLH1*), fork head box, and RUNX3 [21].

Chronic inflammation leads to progressive accumulation of genetic alterations from the normal mucosa, intestinal metaplasia (IM), dysplasia to invasive carcinoma. In a long-term prospective Italian study, 69% of HGD progressed to GC, suggesting the presence of a molecular pathway developing in dysplastic lesions leading to GC onset [19]. IM is characterized by a higher mutation rate in DNA compared to normal gastric mucosa, it shows somatic copy number alterations (sCNAs) in 12.5% of cases and it shows higher levels of DNA methylation than normal mucosa [22]. With regards to dysplastic lesions, microsatellite instability (MSI)/defective mismatch repair (dMMR) profile, *CDH1* inactivation (leading to loss of E-cadherin immunostaining), *HER2* gene amplification and protein overexpression, aberrant p53 expression, increase from LGD to HGD, indicating that they are early drivers of carcinogenesis [23]. Interestingly, a relatively higher prevalence in PD-L1 positivity was observed among gastric dysplastic lesions compared to invasive carcinoma [24].

A less common environmental factor involved in gastric carcinogenesis is Epstein–Barr virus (EBV) infection. EBV infection is more common in men, in the Asian population, it is the highest in proximal tumors and those arising in gastric stump, and it is associated with lymphoepithelioma-like histotype [25]. EBV promotes carcinogenesis through DNA methylation of a series of tumor suppressor genes, resulting in uncontrolled cell growth and in promoting a pro-inflammatory environment. In particular, EBV infection is associated with *PIK3CA* mutation, inactivating mutations of *ARID1A*, and *BCOR* (encoding an anti-apoptotic protein) mutations. Recurrent *JAK2* and *ERBB2* amplification are observed whereas only rare *TP53* mutations of cases are described. In all EBV-related cancers, *CDKN2A* (*p16*^*INK4A*^) promoter hypermethylation is described.

Autoimmune gastritis (AIG) is an autoimmune-mediated disease affecting the parietal cells in the body-fundus, which are the target of serum auto-anti-parietal cell (PCA) and anti-intrinsic factor antibodies [26]. AIG prevalence is significantly higher in middle-age females and may coexist with other autoimmune diseases, such as Hashimoto’s thyroiditis, insulin-dependent diabetes and vitiligo. An association with *HP* infection has been reported, due to molecular mimicry between *HP* and structural proteins of the parietal cells such as the gastric H+/K+ATPase [27]. Epidemiological studies providing data on AIG in the general population are lacking, due to the high rate of asymptomatic or pauci-symptomatic disease in early stages and frequent incomplete (lack of biopsies from the gastric body) mucosal sampling in patients undergoing gastroscopy. Prevalence of AIG has been estimated to be ~ 0.5–4.5% globally, increasing with age from 2.5% in the third decade to 12% in the eighth decade [28]. Interestingly, a few case series described AIG in pediatric patients affected by autoimmune disease [28]. With progressive loss of parietal cells and atrophy of oxyntic mucosa compartment, hydrochloric acid and intrinsic factor levels decrease, leading to stimulation of gastrin-producing cells and enterochromaffin-like (ECL) cells. ECL cell hyperplasia, through linear and micronodular phases, can advance to type 1 gastric neuroendocrine tumor (NET) [28].

Gastric mucosal atrophy may alter the gastric microbiota promoting a microenvironment (“cancerization field”) prone to the development of GC. However, a recent study found that the risk of GC, in naïve *HP*-negative AIG patients, is not increased [29]. The role of the atrophy-modulated gastric microbiota and its likely synergy with HP-induced inflammation in promoting the GC-prone microenvironment deserves further investigation.

Gastric MALT lymphoma is a low-grade lymphoma arising in the gastric mucosa driven by chronic *HP* infection. Gastric MALT lymphoma may regress with *HP* eradication while, in untreated patients, it can turn into extranodal-diffuse large B-cell lymphoma (eDLBCL), a high-grade lymphoma. Gastric MALT lymphoma can also be associated with hepatitis B virus, human immunodeficiency virus (HIV), EBV and human T-cell lymphotropic virus type 1 (HTLV-1) [30]. Inflammatory changes including production of a proliferation inducing ligand (APRIL), a member of the tumor-necrosis factor (TNF)-family, by macrophages, have been associated with lymphoma development [31]. In addition, *HP* can translocate the CagA protein directly into B-cells resulting in extracellular signal-regulated kinase activation and *Bcl-2* expression up-regulation, leading to apoptosis inhibition. Normal B cells are transformed to malignant clone via three chromosomal translocations: t(11;18) (q21;q21), t(1;14)(p22;q32), and t(14;18)(q32;q21), which lead to the activation of NF-κB, which plays a role in immunity, inflammation, and cancerogenesis [32].

## Small bowel inflammation and tumorigenesis

In the small bowel, the inflammation-dysplasia-carcinoma sequence is less well-characterized than in the other gut organs, due to the rarity of primary small intestinal adenocarcinomas and to the well-known technical-endoscopic issues in endoscopically exploring this intestinal tract. Nevertheless, a few immune-inflammatory disorders, including inflammatory bowel diseases (IBDs), celiac disease, as well as long-standing ileostomy, have been consistently associated with increased small bowel cancer risk [33].

In a recent population-based cohort study of patients with IBD diagnosed in Norway and Sweden from 1987 to 2016, the standardized incidence ratio of small bowel adenocarcinoma (SBA) was increased by more than eightfold in Crohn’s disease [34]. Importantly, in this investigation the first year of follow-up was excluded to reduce reverse causality, which may explain, at least in part, the excess risk estimates found during early follow-up. The highest SBA risks were found among patients with Crohn’s disease diagnosed before 40 years (often with a long disease duration before SBA diagnosis), those displaying stricturing behavior or those with inflammatory disease limited to the small bowel. Indeed, most SBAs associated with Crohn’s disease have been found in areas involved by active inflammation, which likely drives cancer development, and they have been found to be associated with metaplastic and/or dysplastic (conventional or non-conventional) mucosal changes, that often share with the adjacent cancer, the expression of gastro-pancreato-biliary markers [35–37]. On the other hand, small bowel resection and use of salicylates for more than two years seem to protect against SBA in patients with Crohn’s disease [38]. Some molecular alterations, such as *IDH1* gene mutations, are enriched in SBAs associated with Crohn’s disease compared to sporadic cases, while *APC* mutations seem to be rarer in the former [39, 40].

In addition, Yu et al. reported that the risk of SBA is also increased (about twofold) in ulcerative colitis patients, where it was strongly associated with extensive disease. However, the relationship between ulcerative colitis and SBA is still uncertain, as a recent meta-analysis of 26 observational studies failed to find a significantly increased risk of SBA in ulcerative colitis [41]. Some subgroups of IBD patients may also have an increased risk of developing lymphoma; however, no association with disease severity was found [42]. Due to the low absolute risk of small bowel neoplasms, active surveillance of the small intestine is currently not recommended in IBD patients.

Interestingly, Yu et al. also found that the standardized incidence ratio of small intestinal NETs was increased (about twofold) both in Crohn’s disease and in ulcerative colitis patients, in the latter likely confined to patients with extensive colitis [34]. However, it should be noted that small intestinal NETs are usually not detected at sites of active inflammation, and they are often incidental findings in IBD surgical resection specimens. Therefore, the causal relationship between small intestinal mucosal inflammation and NET development remains to be elucidated. Interestingly, in IBD and NET patients, common patterns of microbiome composition (e.g., depletion of *Faecalibacterium prausnitzii*, which plays a role in modulating the immune system and to protect the gut barrier integrity by the production of butyrate) have been observed [43]; notwithstanding this observation, the role of intestinal microbiota in NET development requires further investigation. A recent investigation by Massironi et al. found that 13% of duodenal NETs were associated with duodenal gastric surface metaplasia, defined as the replacement of the normal duodenal epithelial cells with cells that resemble gastric foveolar epithelium [44]. Once again, this finding indirectly suggests that chronically inflamed microenvironment may play a role in the development of a subset of duodenal NETs, as duodenal gastric metaplasia is often related to chronic inflammation of the duodenal mucosa, due to abnormally high production of gastric acid triggered by *HP* infection or to drug-induced injury, celiac disease or Crohn’s disease. In addition, duodenal gastric metaplasia harboring *KRAS* or *GNAS* mutations may represent a precursor lesion of duodenal adenoma and adenocarcinoma [45]**.**

Another immune-mediated intestinal disorder, celiac disease, has also been found to be associated with an increased overall cancer risk (essentially confined to celiac individuals diagnosed after age 40) compared to the general population [46–48]. An increased risk of SBA, hemato-lymphoid (intestinal and non-intestinal) neoplasms, in particular enteropathy-associated T cell lymphoma (EATL), as well as other GI malignancies (e.g., pancreatic carcinoma) have also been described in celiac patients. Although previous studies reported a pooled odds ratio of 14.4 for SBA in celiac disease [49], a recent Swedish nationwide cohort of celiac individuals, accurately designed to reduce the risk of detection bias in the peri-diagnostic period, estimated the hazard risk of SBA and small bowel adenomas to be between 3.05 and 5.73 in celiac individuals in comparison with matched reference individuals, and the SBA risk was higher in the first 10 years of follow-up [50]. In the latter study, “mucosal healing” (defined as Marsh 0–2) after gluten-free diet was associated with a lower, albeit not statistically significant, risk of SBA in celiac individuals, suggesting the role of small bowel inflammation in the pathogenesis of SBA. It should be noted, however, that the absolute risk of SBA in celiac patients is low (0.06% in Emilsson’s study), thus not implying a need for surveillance. The hypothesis that SBAs arise from the classic “adenoma-to-carcinoma sequence” in celiac patients is still highly debated, due to the rarity of adenomatous dysplastic growths adjacent to the invasive adenocarcinoma [36]. Importantly, patients with celiac disease associated-SBA showed higher rates of MSI/dMMR and more favorable prognosis compared to patients with sporadic SBAs or SBAs associated with Crohn’s disease [51, 52]. On the contrary, EATL, a high-grade lymphoma typically associated with celiac disease, is a very aggressive disease, generally with an ominous outcome. While no risk factors for the development of SBA, have been identified, apart from the higher age at diagnosis of celiac disease, likely indicative of diagnostic delay, EATL may be preceded by type 2 refractory celiac disease. The latter is a rare form of complicated celiac disease characterized by villous atrophy and a monoclonal expansion of immunophenotypically abnormal intraepithelial T lymphocytes, which accumulate in the intraepithelial compartment driven by increased production of the potent anti-apoptotic and proliferative properties of IL-15 [53]. Moreover, rare cases of monomorphic epitheliotropic intestinal T cell lymphomas have been described in celiac patients [54]. Several studies provided evidence that strict gluten-free diet may decrease cancer risk and mortality, whereas non-adherence and/or non-responsiveness to a gluten-free diet may result in persistent mucosal chronic inflammation, which, eventually, might promote the development of lymphoma or carcinoma [47, 55].

Finally, intestinal T cell lymphomas have been described in patients with non-celiac enteropathies, such as autoimmune enteropathy [56], while patients with common variable immunodeficiency have been reported to be at increased risk for gastric adenocarcinoma and intestinal lymphomas, the latter usually arising in the setting of nodular lymphoid hyperplasia [57, 58].

## Large bowel inflammation and tumorigenesis

Patients with IBD are at high risk for developing dysplasia and colorectal cancer (CRC) through an inflammation-dysplasia-carcinoma sequence [59]. IBD, encompassing Crohn’s disease and ulcerative colitis, is a chronic inflammatory disorder of the GI tract, caused by a dysregulated inflammatory and immune response in genetically susceptible individuals. An altered gut microbiome (dysbiosis), as well as other environmental factors, play an important role in triggering and perpetuating inflammation. Patients have a relapsing and remitting disease course, often with bloody diarrhea and abdominal pain in moments of active disease, interspersed with periods of remission. Individuals with IBD are at an increased risk of developing neoplasia, in particular CRC, but also SBA, intestinal lymphoma and anal cancer, as well as tumors in extraintestinal sites. Population-based studies have shown an estimated risk of CRC 2- to threefold that of the general population in ulcerative colitis patients [60], and Crohn’s disease patients appear to have a similar increased risk [60]. IBD-related CRCs often show peculiar histotypes (such as mucinous and signet ring carcinomas [61]), they are more often proximal in location and are high-grade malignancies with poorer overall survival compared to sporadic CRC.

Sporadic CRC follows the adenoma-carcinoma sequence, while IBD related CRC has been shown to follow the ‘inflammation–dysplasia–carcinoma’ sequence. In this context, inflammation plays a crucial role as the relapsing–remitting inflammatory nature of disease causes epithelial destruction and regeneration [62]. Chronic inflammation is involved in tumorigenesis through various mechanisms, including oxidative stress with DNA damage, abnormal immune response and involvement of the gut microbiota. In particular, epithelial proliferation induced by mucosal regeneration increases mutational burden and the selection of mutated clones. Mutagenesis is in part induced and driven by inflammation, by production of pro-inflammatory cytokines (IL-1, IL-6, TNF-α) and chemokines and the generation of reactive oxygen species and lipid peroxidation leading to increased inflammation-induced oxidative DNA damage (with accumulation of mutations). The inflammation-induced activation of nuclear transcription factors (NF-kB and STAT3) which perpetuate inflammation and promote carcinogenesis via the loss of the p53 tumor suppressor gene leads to unchecked cell growth and inhibition of apoptosis with increase of cytokine-mediated DNA damage. Indeed, TP53 mutations have been observed in non-dysplastic epithelial cells in inflamed mucosa underlining how inflammation plays an initial and pivotal role in the development of IBD-related CRC.

Inflammation, not only drives the initiation of cancer but it is also involved in disease progression and this can be observed from a morphologic point of view also. The cancerogenic sequence therefore starts with intestinal mucosa which has been genetically modified by chronic active inflammation and on this basis, the sequence from LGD to HGD to IBD-related CRC is initiated [63].

The standardized classification system of IBD-related dysplasia was introduced by Riddell et al. in 1983 dividing dysplasia into categories, including (indefinite for dysplasia) LGD, HGD and invasive carcinoma [64]. Conventional (or intestinal type) dysplasia is the most well-recognized form of dysplasia, and the identification and grading of dysplasia in IBD (according to Riddell) is the cornerstone of management of these patients. Recently, SCENIC (Surveillance for Colorectal Endoscopic Neoplasia Detection and Management in Inflammatory Bowel Disease Patients) guidelines [65] have stressed another important feature of dysplasia, specifically whether it is endoscopically visible or invisible. These endoscopic features guide patient management, as polypoid/visible dysplasia (even HGD) can be treated endoscopically while colectomy is the treatment of choice for flat/invisible dysplasia (especially in HGD or multifocal LGD). Endoscopic surveillance of IBD patients is therefore fundamental for recognizing early lesions which can be treated conservatively and which reduce neoplastic risk in these patients.

While conventional (intestinal type) dysplasia has garnered, up till now, most interest, new non-conventional patterns have been collected and described in recent years. Seven morphologic categories have been described including hypermucinous dysplasia (the most common), goblet cell-deficient, crypt cell dysplasia, increased Paneth cell differentiation and serrated lesions. Recognition of these non-conventional dysplastic (NCD) lesions is important as they are common in IBD patients with dysplasia (up to 33% of dysplastic lesions are non-conventional) and IBD patients harboring CRC (45% of IBD-associated CRC had associated NCD lesions in one series) [66, 67]. NCD lesions may be seen either adjacent to CRC or within the same segment, they may be found associated with conventional dysplasia and, despite their low-grade appearance, they are associated with high grade (poorly differentiated) CRC. Furthermore, new studies have shown that NCD lesions (especially hypermucinous, goblet cell-deficient, and crypt cell dysplasia), often graded as LGD, have a higher rate of aneuploidy, *KRAS* mutations and appear to have a higher risk of progression to HGD/CRC compared to conventional dysplasia. These NCD lesions are more frequently flat/invisible (40% in NCD lesions compared to 18% for conventional dysplastic lesions) making endoscopic surveillance and treatment ever more important. An extremely recent contribution has shown that increased histologic inflammation is an independent risk factor for NCD, showing an increased cumulative inflammation burden compared to non-dysplastic UC patients [68].

Risk factors for malignancy have been identified in IBD patients, and the most important are correlated with inflammation. In particular long-standing IBD has been shown to be correlated with increased cancer risk. Older series reported CRC risk as high as 15% in patients with 30 years of active disease [69], while more recent estimates, based on large population-based studies and meta-analyses, identify lower (though absolutely not negligible) percentage risks [70]. Furthermore, active disease and severity of inflammation increase the risk of dysplasia and CRC as well as disease extent [71]. Other culprits of increased CRC risk in IBD patients include primary sclerosing cholangitis (threefold increase), family history of CRC (twofold increase) and younger onset-IBD (likely attributable to longer disease duration) [72].

Recent contributions have also investigated the effect of gut microbiome on IBD-related (and sporadic) CRC and are gaining an increasing level of interest [73]. Various theories concerning bacterial involvement in IBD-related CRC have been proposed, where dysbiosis is probably an active participant in the inflammation-dysplasia-cancer sequence. The passage of gut bacteria (such as *E. coli* and enterotoxigenic *Bacteroides fragilis)* from the lumen into the subepithelial tissue, through mucosa barrier disruption, sustains inflammation, with an increase in pro-inflammatory and pro-carcinogenic mediators increasing the risk of developing CRC.

## Conclusions

**Fig. 2:**
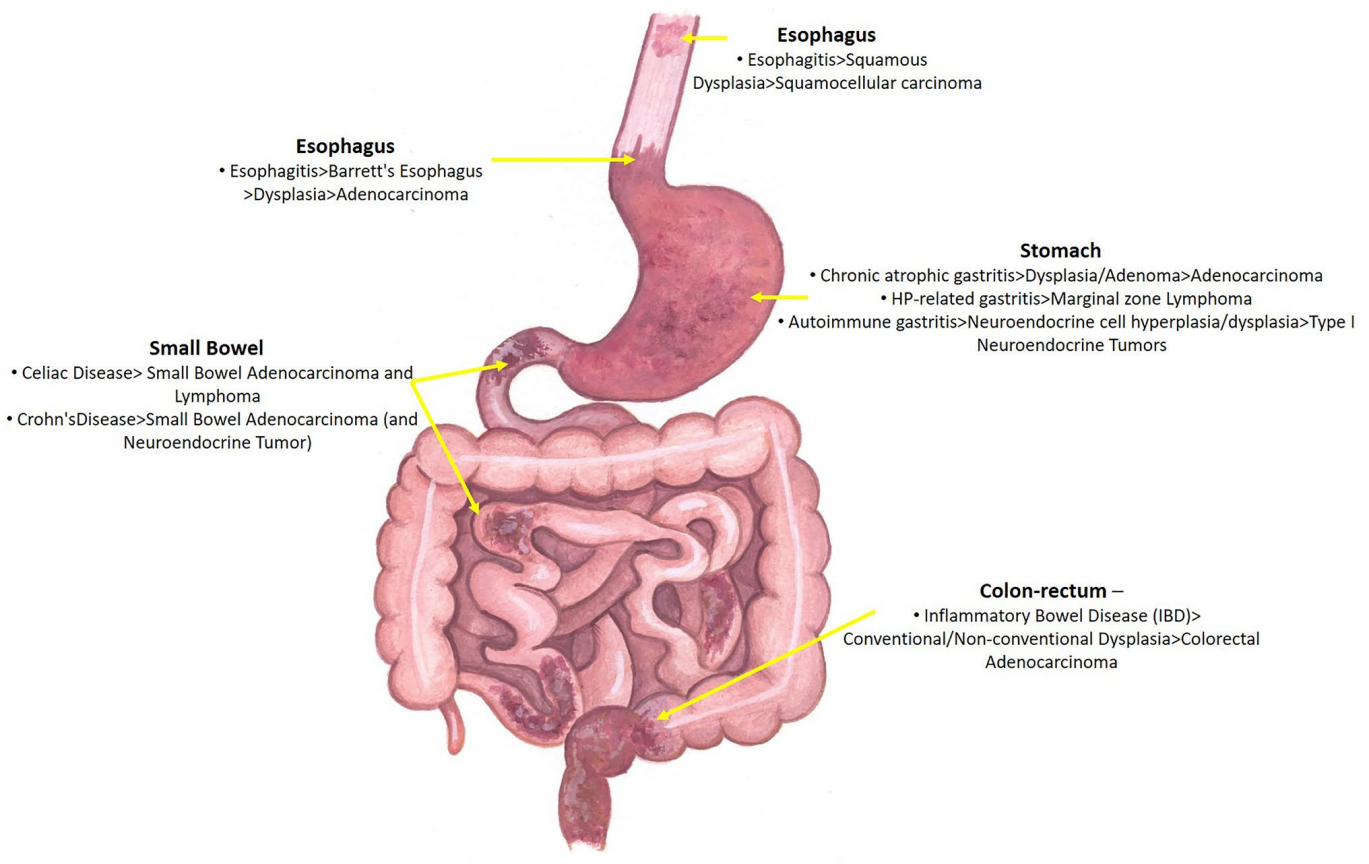
A schematic summary of the principal inflammatory conditions which predispose to cancer development in various sites along the gastrointestinal tract

The etiologically heterogeneous inflammatory disorders affecting the diverse organs of the tubular GI tract predispose to diverse epithelial and non-epithelial neoplasms, as summarized in Fig. 2. An inflammation-dysplasia-carcinoma sequence has been well characterized in some conditions, such as in the BE-associated EAC, HP-gastritis-related GC or in IBD-related CRCs, whereas the tumorigenic processes are poorly known in other sites, such as the small bowel. The identification and modulation of cancer-inducing molecular mechanisms and gut dysbiosis may open the door for treatment and prevention of GI neoplasms (e.g., IBD-related CRC) in the future.

## Data Availability

Not applicable.
